# The Swine Erysipelas Vaccine SER-ME Effectively Protects Pigs against Challenge with the *Erysipelothrix rhusiopathiae* M203/I257 SpaA-Type Variant

**DOI:** 10.3390/vetsci9080382

**Published:** 2022-07-26

**Authors:** Misako Morimoto, Atsushi Kato, Kotoe Nogami, Yuta Akaike, Takaaki Furusawa, Hiroe Kojima, Chihiro Sasakawa

**Affiliations:** 1 Nippon Institute for Biological Science, 9-2221-1 Shin-machi, Ome 198-0024, Tokyo, Japan; 2 Biomedical Science Association, 2-20-8-3F Kamiosaki, Shinagawa-ku, Tokyo 141-0021, Japan; 3 Medical Mycology Research Center, Chiba University, 1-8-1 Inohana, Chiba 260-8673, Chiba, Japan

**Keywords:** *Erysipelothrix rhusiopathiae*, SpaA, swine erysipelas, variant, vaccine efficacy

## Abstract

**Simple Summary:**

Swine erysipelas caused by the *Erysipelothrix rhusiopathiae* occurred frequently among pigs in Japan mostly by the serovar 1a variant featured by two amino acids in the surface protective antigen A protein. To determine if current vaccines are effective against the variant infection in pigs, one inactivated vaccine, SER-ME containing serovar 2a as the immunogen, was representatively evaluated. All vaccinated pigs survived without any apparent clinical signs after lethal challenge with the Fujisawa reference strain or the variant. This indicates that the serovar 2a immunogen of SER-ME vaccine effectively protects pigs against the *E. rhusiopathiae* variant and was not related to the emergences of the variant.

**Abstract:**

*Erysipelothrix rhusiopathiae* causes swine erysipelas (SE). Sporadic SE outbreaks in Japan are mostly caused by the *E. rhusiopathiae* serovar 1a variant featured by methionine (M) and isoleucine (I) at amino acid positions 203 and 257 of the surface protective antigen (Spa) A protein (M203/I257 SpaA-type). To determine if current vaccines are effective against infection with this variant in pigs, one representative inactivated vaccine, SER-ME (containing *E. rhusiopathiae* serovar 2a), was evaluated. All vaccinated pigs survived without any apparent clinical signs after lethal challenge with the Fujisawa reference strain or the variant. This indicates that the SER-ME vaccine effectively protects pigs against the infection of *E. rhusiopathiae* M203/I257 SpaA-type variant. Current vaccines in Japan, including SER-ME, suggest that outbreaks in Japan are unlikely caused by vaccine failure.

## 1. Introduction

*Erysipelothrix rhusiopathiae*, which causes swine erysipelas (SE), is a zoonotic bacterium that causes economic loss in pig farms. Infection with *E. rhusiopathiae* in pigs can be classified as acute, subacute, or chronic. The main acute sign is sudden death from sepsis or miscarriage. Subacute signs include fever and anorexia, as well as characteristic skin lesions, such as urticaria (diamond skin). Common chronic signs are arthritis, lymphadenitis, and endocarditis [1].

*E. rhusiopathiae* have been divided 16 serovars; 1, 2, 4–6, 8, 9, 11, 12, 15–19, 21, and N. Serovar N does not react with serovar-specific antigens [2,3]. Among these serovars, *E. rhusiopathiae* of serovar 1 and 2 are the major pathogens worldwide and can be further subdivided into 1a or 1b, and 2a or 2b [4]. Serovar N has a possibility to be transitioned to other serovars in the future. A number of surface proteins, such as choline-binding proteins (Cbp) B, glyceraldehyde-3-phosphate dehydrogenase (GAPDH), *E. rhusiopathiae* surface protein (RSp) A, and surface protective antigen (Spa) A, B and C, has been identified as protective antigens of *E. rhusiopathiae* [5]. Among these surface proteins, SpaA is most investigated [6]. The carboxyl-terminal region of Spa protein has structural homology to the Cbps of *Streptococcus pneumonia* that contains glycine–tryptophan repeats [7,8], and Spa adheres to the phosphorylcholine on endothelial cells via this region [9]. The aminoacyl-terminus of the Spa protein has a variable region that is required for the induction of protective immunity [4]. Protective immunity is not restricted by serovars, and different levels of cross-protection beyond serovars have been observed [10,11].

Six positions are known as a single nucleotide polymorphisum (SNP) site in the hypervariable region of *spaA* gene [12]. Until recently four SpaA variants, in which amino acid change(s) occurred on variable region of *spaA* gene, were found in Japan [13,14]; these variants are a variant having a single isoleucine to methionine (M) change at position 203 (M203 SpaA-type), a variant having double changes, isoleucine to methionine at position 203 and lysine to isoleucine (I) at position 257 (M203/I257 SpaA-type), a variant having a glutamine to aspartic acid (D) change at position 242 in addition to M203 change (M203/D242 SpaA-type), and a variant having single aspartic acid to alanine (A) change at 195 (A195 SpaA-type). Since 2008, erysipelas has occurred frequently among pigs in Japan. Importantly, the outbreaks in Japan from 2008 to 2011 were caused mainly by serovar 1a (95.2%), and among these, 96.2% was M203/I257 SpaA-type variant [13]. Predominance of this variant (74.6%) was still observed in strains isolated during the outbreak in Japan from 2012 to 2019 [15]. This indicates that the serovar 1a M203/I257 SpaA-type variant of *E. rhusiopathiae* is the major causative strain of SE as ever in Japan. In a previous study, we found that serovar 1a strains have advantages on growth in liquid culture compared with serovar 2a and showed more virulent phenotype than serovar 2a in mice and pigs [16]. However, the reason why the *E. rhusiopathiae* serovar 1a M203/I257 SpaA-type variant has become dominant in the field in Japan remains unknown. A live attenuated SE vaccine licensed in Japan containing *E. rhusiopathiae* Koganei 65-0.15 (serovar 1a) has been shown to protect pigs from the SpaA variant strain [17].

In this study, we used SER-ME containing *E. rhusiopathiae* Tama-96 (serovar 2a) as a representative of current inactivated vaccines to investigate whether it was effective for protecting pigs against infection with the Fujisawa reference strain (serovar 1a) or the M203/I257 SpaA-type variant.

## 2. Materials and Methods

### 2.1. Strains, Culture Conditions and Colony-Forming Unit (CFU) Assay

The Fujisawa strain was used as the reference strain of *E. rhusiopathiae* [18]. One of the major SpaA-type variants having M203/I257 SpaA-type, 2012 Miyazaki variant that was isolated previously [15,16], was representatively used. The Fujisawa strain and 2012 Miyazaki variant belong to serovar 1a variant. The Marienfelde strain was used for the growth agglutination (GA) test as the live antigen (*See below*). Three strains were prepared from the glycerol stock in the brain heart infusion (BHI) medium as previously described [16].

Fujisawa strain and 2012 Miyazaki variant were cultured at 37 ◦C in the tryptose phosphate broth (TPB) medium. Marienfelde strain was cultured in BHI medium [16]. Colony-forming units (CFU) of Fujisawa strain and 2012 Miyazaki variant were measured by counting the colonies on TPB agar plates as previously described [15].

For the isolation of *E. rhusiopathiae* from experimentally infected pigs, blood, and organ homogenates were cultured on BHI agar plate with 400 µg/mL of kanamycin and 25 µg/mL of gentamycin as previously described [13]. Once colonies were observed, DNA was extracted as previously described [19], and SpaA PCR was performed to determine if it was the challenging strain (*See next section for details*).

### 2.2. Sequencing of SpaA Genes

A 432 bp-DNA fragment in the 5′ half region of the *spaA* gene was amplified by the polymerase chain reaction, and amplified DNA was used for the sequence analysis as previously described [10,20] to confirm the challenging strain of *E. rhusiopathiae* in pigs. Different nucleotides and amino acids between *E. rhusiopathiae* Fujisawa strain and 2012 Miyazaki variant (serovar 1a, M203/I257 SpaA-type) are shown in Table 1.

### 2.3. Vaccine Used for Immunization

SER-ME is an inactivated SE vaccine that is produced from the *E. rhusiopathiae* Tama-96 strain. It was obtained from Nisseiken Co. Ltd. (Ome, Japan) and was used for pig immunizations according to the manufacturer’s dosage and administration. Serovar of Tama-96 strain was identified as serovar 2a by agar gel precipitation tests using type-specific rabbit antisera [13].

### 2.4. Vaccination and Challenge Exposure in Pigs

The animal experiment was conducted by veterinarians according to the protocol approved by the Experimental Animal Care and Use Committee of Nippon Institute for Biological Sciences (NIBS). The humane endpoint was applied if the pigs were deemed unable to stand and drink freely due to the challenge.

Sixteen pigs (LWD: Landrace, White Yorkshire × Duroc), which were over 4 weeks old, were purchased from a conventional farm free from SE where no pigs were vaccinated against the disease. The ELISA and GA antibody titer of the sow were measured (*See below*), and the piglets antibody titer was estimated and determined to be free from SE. Experiments were performed following the quarantine acclimatization (1 week). Pigs were divided into four groups, and each group was comprised of four pigs assigned randomly, regardless of male or female. Pigs in groups 1 and 3 were vaccinated twice, 3 weeks apart, with 0.5 mL of SER-ME (Lot.1) according to the manufacturer’s dosage and application. While those in groups 2 and 4 were not vaccinated and served as the non-immunized controls. Adverse events following the vaccination were observed for 7 days. Pigs in groups 1 and 2 were challenged with 5.0 × 10^7^ CFU/head of the *E. rhusiopathiae* Fujisawa strain, while those in groups 3 and 4 were challenged with 7.8 × 10^7^ CFU/head of the *E. rhusiopathiae* 2012 Miyazaki variant. This challenge dose was determined by referring to our previously study [14].These challenges were administrated subcutaneously.

Clinical responses were observed daily for 14 days after the challenge. Fever, arthritis, and depression were counted as +1. Urticaria was counted as +1, +2, or +3, depending on its severity: +1, urticarial lesions those only observed around the inoculation site; +2, those around the cervical region; and +3, systemic erythema. Death or the humane endpoint was scored as +7. These clinical scores followed our previous study [16].

### 2.5. Collection of Blood and Organs Samples from Experiment Pigs

Serum samples were taken from pigs at five time points: on day 0, just before the primary vaccination; on day 14, 2 weeks after the primary vaccination; on day 21, just before the booster vaccination; on day 35, just before the challenge; and on day 49, at the end of the study. Blood samples were collected at 3 days after the challenge and were diluted for 10- and 100-fold with phosphate buffered saline. Portions (0.1 mL) of each dilute were spread onto two agar plates. Pigs that died (including humane end point) by challenging of *E. rhusiopathiae* were necropsied as soon as we noticed. Pig organs, namely heart, liver, spleen, kidney, lung, synovial fluid, and mandibular lymph nodes, were collected to isolate *E. rhusiopathiae*.

### 2.6. Measuring Antibody Titers in Pigs (ELISA and GA Titers)

Anti-*E. rhusiopathiae* antibodies in serum samples were detected with the CIVTEST SUIS SE/MR indirect ELISA kit (HIPRA, Amer, Spain), on which the specific antigen (details not disclosed from the manufacturer) of *E. rhusiopathiae* was coated, based on the sample-to-positive ratio according to the manufacturer’s instruction. Additionally, the GA test was basically performed according to standard procedures [22] with minor modifications by us [16].

### 2.7. Statistical Analyses

Statistical analyses were performed using Student’s *t*-test to compare groups. Data are presented as the mean ± standard deviation (SD) for four samples per group. *p* < 0.01 was considered statistically significant.

## 3. Results

### 3.1. Vaccination and Serum Titers for E. rhusiopathiae

SER-ME, an SE vaccine that is licensed in Japan, was used for the vaccinations. SER-ME is an oil-adjuvanted, inactivated SE vaccine containing the Tama-96 strain (serovar 2a) of *E. rhusiopathiae* as the immunizing component. Sixteen nearly 5-week-old pigs were used in this study. Half of the pigs (8/16) were vaccinated, and the rest were unvaccinated and used as controls. At the primary vaccination, four of the eight vaccinated pigs developed a red patch at the injection site, two of the eight pigs had induration at the injection site, and two of the eight pigs experienced transient fever (>40.5 °C) (Supplementary Table S1). Three weeks after the primary vaccination, the same eight pigs (groups 1 and 3) were given a single booster vaccine. None of the pigs exhibited adverse events following the booster vaccination except two of the eight pigs had induration (Supplementary Table S1).

**Figure 1:**
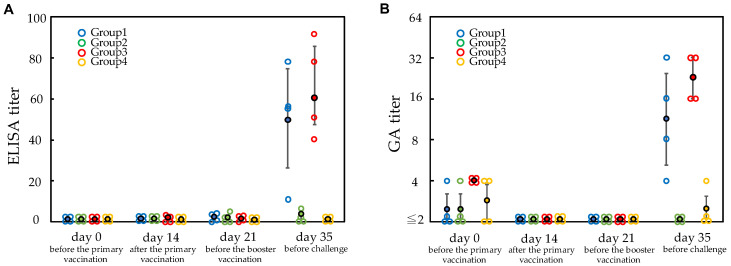
**Antibody titers against*Erysipelothrix rhusiopathiae*measured using ELISA and the growth agglutination (GA) tests.** The *X*-axis shows the blood collection at four time points; on day 0, just before the primary vaccination; on day 14, 2 weeks after the primary vaccination; on day 21, just before the booster vaccination; on day 35, just before the challenge. The *Y*-axis shows the antibody titers. Each antibody titer of pigs (open circle) and mean antibody titer (closed circle) of each group are shown. Each group of pigs is shown in separate color. Vertical line on the mean antibody titer indicates the ±SD error bar. (**A**) ELISA titers. (**B**) GA antibody titers.

Anti-*E. rhusiopathiae* titers in pig sera were measured using a commercially available ELISA kit (Figure 1A). Serum titers measured using a commercially available ELISA kit were negative in *E. rhusiopathiae*. The pigs in groups 1 and 3, which were vaccinated, showed no significant increase in anti-*E. rhusiopathiae* titers at 3 weeks after the primary vaccination but did exhibit a marked increase at 2 weeks after the booster vaccination. The pigs in groups 2 and 4, which were unvaccinated, showed no increase in anti-*E. rhusiopathiae* titers during the immunization period. GA tests were performed to confirm the results of ELISA tests. While GA titers (>8) were not elevated at 3 weeks after the primary vaccination in groups 1 and 3, they were increased at 2 weeks after the booster vaccination. There were no increases in GA titers in groups 2 and 4 (Figure 1B). Anti-*E. rhusiopathiae* titers measured by ELISA and GA tests showed good agreement and were very high in all pigs in groups 1 and 3 after vaccination, although one of the eight pigs had somewhat lower titers than the other seven, which was belonged to group 1 (*See day 35 of* Figure 1).

### 3.2. Protection of Pigs against E. rhusiopathiae Challenge

**Figure 2:**
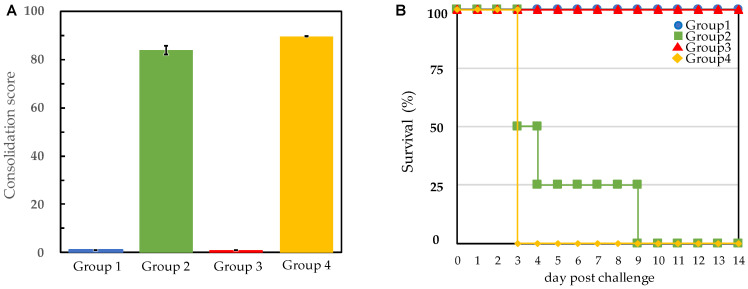
**Pig protection test against two different strains of*Erysipelothrix rhusiopathiae*following vaccination with SER-ME.** (**A**) Inclusive sum of clinical scores during the experimental period (14 days) are shown. Group 1 and 3 were vaccinated groups. Group 2 and 4 were unvaccinated groups as the control. Group 1 and 2 were challenged with Fujisawa reference strain. Group 3 and 4 were challenged with 2012 Miyazaki variant. Clinical signs scored are shown in Materials and Methods (*See* Section 2.4). Vertical line on each bar graph shows the ±SD error bar. (**B**) Pigs were observed daily to detect clinical signs of disease for 14 days after challenge (*Y*-axis line). The number of surviving pigs is indicated in *X*-axis line.

After serum antibody titers against *E. rhusiopathiae* were confirmed, challenge tests were performed using the Fujisawa reference strain or the M203/I257 SpaA-type 2012 Miyazaki variant, both of which belong to serovar 1a of *E. rhusiopathiae* (Table 2). The results of daily observations of clinical signs are summarized in Table 2 and Figure 2A. Vaccinated pigs in groups 1 and 3 showed no apparent clinical signs regardless of the *E. rhusiopathiae* strain used during the challenge, indicating that vaccination protected pigs not only from the Fujisawa reference strain but also from the newly emerged M203/I257 SpaA-type Miyazaki variant. However, all the unvaccinated pigs in group 2 and 4 developed SE. In group 2, two of the four pigs died (including the humane endpoint, the same shall apply hereafter) on the third day after Fujisawa strain challenge, one pig died on the fourth day, and the remaining pig died on the ninth day (Figure 2B). In group 4, all four pigs died on the third day after challenge. The sums of the clinical scores were not significantly different (*p* < 0.01) between unvaccinated pigs challenged with the Fujisawa strain (group 2) versus the Miyazaki variant (group 4).

### 3.3. Isolation of E. rhusiopathiae from Challenged Pigs

Pig organs and blood samples were collected at the time of autopsy or death to microbiologically confirm vaccine effectiveness. Specifically, organ specimens were obtained from the heart, lungs, liver, kidneys, spleen, and mandibular lymph nodes. In vaccinated groups 1 and 3, no *E. rhusiopathiae* cells were detected in organ samples (Supplementary Table S2) or blood (Table 2), indicating robust vaccine-induced immunity. In unvaccinated groups 2 and 4, however, 10^2^ to 10^7^ CFU/mL of *E. rhusiopathiae* were isolated from bloods. PCR amplification and sequencing analysis of *spaA* gene showed that the isolated *E. rhusiopathiae* strain had identical strain-specific DNA regions as the challenging strain (data not shown). Therefore, there was no contamination of the challenging strain.

## 4. Discussion

In this study, we investigated the efficacy of the SER-ME vaccine, an oil-adjuvanted, inactivated SE vaccine, to determine whether Tama-96 strain (serovar 2a) of *E. rhusiopathiae* in the vaccine was as effective against *E. rhusiopathiae* M203/I257 SpaA-type variant as against the serovar 1a Fujisawa strain. All vaccinated pigs survived and exhibited no clinical signs regardless of the challenge strain. The fact that unvaccinated pigs showed no significant difference in clinical signs after challenge with the Fujisawa strain or the variant suggested that, at least in pigs, both strains are almost equally virulent. Although vaccinated pigs differed in terms of their antibody titers, both vaccinated pig groups were equally protected. This suggested that the immunity conferred by the SER-ME vaccine was similar for the SE caused by *E. rhusiopathiae* serovar 1a M203/I257 SpaA-type variant and that caused by the serovar 1a Fujisawa strain. Based on previous studies, a live attenuated vaccine, Koganei 65-0.15 (serovar 1a) available in Japan, was effective for pigs against the M203/I257 SpaA-type variant [17]. Major live and inactivated SE vaccines used in Japan are sufficiently effective to protect pigs against SE, even that caused by the *E. rhusiopathiae* serovar 1a M203/I257 SpaA-type variant. This strongly indicates that the dominance of this variant in Japan is not simply caused by primary vaccine failure.

*E. rhusiopathiae* exists in the natural environment, including soil and wastewater and in wild animals [23]. It is even found in apparently healthy pigs that serve as subclinical carriers [24]. It is thus important to eliminate the *E. rhusiopathiae* around the pig houses by using good hygiene and intensive vaccination. Although this study suggests that the emergence of the serovar 1a M203/I257 SpaA-type variant in Japan cannot be explained by weak or absent vaccine-induced immunity, overreliance on vaccination may reduce the importance placed on daily hygiene measures. In fact, appropriate vaccination and proper hygiene are both necessary for maximal disease control. It is thought that neglect of one or both of these measures has led to sporadic outbreaks of SE. Considering the wide host range of *E. rhusiopathiae*, transmission via wild animals, such as mice, rats, wild boars, and birds, is important [3,25]. Moreover, it is also useful for pig health to improve the comfort of the breeding environment because pigs exposed to the stress in the pig house can reduce the immunological responses against the pathogens. Since it remains unclear why the M203/I257 SpaA-type variant of *E. rhusiopathiae* is currently the major causative agent in outbreaks, we will further analyze the pathogenicity of the variants.

## Figures and Tables

**Table 1:** ***E. rhusiopathiae*** **strains in the SE vaccines and for pig challenge tests.**

Strains and SpaA-Type Variant (Serovar)	Difference of Nucleotide (Amino Acid) in *spaA* Gene			
	555 (185)	584 (195)	609 (203)	769 (257)
Koganei 65-0.15 (1a)	CCC (P)	GAT (D)	ATT (I)	ATT (I)
Tama-96 (2a)	CCA (P)	GAT (D)	ATT (I)	CTT (K)
Fujisawa (1a)	CCC (P)	GAT (D)	ATT (I)	CTT (K)
2012 Miyazaki, M203/I257 SpaA-type (1a)	CCC (P)	GAT (D)	ATG (M)	ATT (I)

Six positions known as a single nucleotide polymorphisum (SNP) site in the hypervariable region of *spaA* gene are shown [12,21]. Different nucleotides and amino acids (one letter code) relative to the Fujisawa strain are shown in underline.

**Table 2:** **Clinical responses of pigs challenged with** ***E. rhusiopathiae*.**

Group No.	Pig No.	Immunization	Challenge Strain	Presence of Clinical Signs after Challenge				Bacterial Isolation from Blood *^3^
				Pyrexia (°C) *^1^	Erythema *^2^	Depression	Mortality	
1	1	Vaccinated (SER-ME)	Fujisawa (reference)	None	−	−	Survive	Below detection limit
	2			None	−	−	Survive	Below detection limit
	3			None	−	−	Survive	Below detection limit
	4			None	−	−	Survive	Below detection limit
2	5	Unvaccinated control		41.5	+++	+	Dead (Day9)	2.2 × 10^2^
	6			41.4	+++	+	Dead (Day3)	4.9 × 10^5^
	7			41.7	+++	+	Dead (Day3)	2.3 × 10^3^
	8			41.8	+++	+	Dead (Day4)	5.0 × 10^3^
3	9	Vaccinated (SER-ME)	2012 Miyazaki (variant)	None	−	−	Survive	Below detection limit
	10			None	−	−	Survive	Below detection limit
	11			None	−	−	Survive	Below detection limit
	12			None	−	−	Survive	Below detection limit
4	13	Unvaccinated control		41.7	+++	+	Dead (Day3)	8.2 × 10^5^
	14			41.7	+++	+	Dead (Day3)	1.8 × 10^7^
	15			41.7	+++	+	Dead (Day3)	1.2 × 10^6^
	16			41.9	+++	+	Dead (Day3)	9.1 × 10^5^

*^1^ A fever of 40.5 °C and the highest during the 14 days observation period were described. *^2^ Urticaria lesion was scored as +, ++ and +++ depending on its severities described in Materials and Methods. *^3^ Blood was collected 3 days after challenge. CFU/mL is shown. If no colony was found in a plate on which 0.1 mL of undiluted blood was spread, they are shown as “Bellow detection limit” and are equal to <1 × 10^1^ CFU/mL.

## Data Availability

Majority of data generated during this study is included in this published article and its supplementary tables. Some datasets that are not shown are available from the corresponding author on reasonable request.

## Supplementary Materials

The following supporting information can be downloaded at: https://www.mdpi.com/article/10.3390/vetsci9080382/s1, Table S1: Adverse events following the vaccination, Table S2: Detection of *E. rhusiopathiae* cells in organs of pigs.

## References

[1] Opriessnig T., Coutinho A.T., Zimmerman J.J., Karriker A.L., Ramirez A., Schwartz J.K., Stevenson W.G., Zhang J. (2019). Erysipelas. Diseases of Swine.

[2] To H., Someno S., Nagai S., Koyama T., Nagano T. (2010). Immunization with Truncated Recombinant Protein SpaC of *Erysipelothrix rhusiopathiae* Strain 715 Serovar 18 Confers Protective Immunity against Challenge with Various Serovars. Clin. Vaccine Immunol..

[3] Opriessnig T., Forde T., Shimoji Y. (2020). *Erysipelothrix* Spp.: Past, Present, and Future Directions in Vaccine Research. Front. Vet. Sci..

[4] Imada Y., Goji N., Ishikawa H., Kishima M., Sekizaki T. (1999). Truncated Surface Protective Antigen (SpaA) of *Erysipelothrix rhusiopathiae* Serotype 1a Elicits Protection against Challenge with Serotypes 1a and 2b in Pigs. Infect. Immun..

[5] Zhu W., Wu C., Kang C., Cai C., Wang Y., Li J., Zhang Q., Sun X., Jin M. (2018). Evaluation of the Protective Efficacy of Four Newly Identified Surface Proteins of *Erysipelothrix rhusiopathiae*. Vaccine.

[6] Borrathybay E., Gong F.J., Zhang L., Nazierbieke W. (2015). Role of Surface Protective Antigen A in the Pathogenesis of *Erysipelothrix rhusiopathiae* Strain C43065. J. Microbiol. Biotechnol..

[7] Rosenow C., Ryan P., Weiser J.N., Johnson S., Fontan P., Ortqvist A., Masure H.R. (1997). Contribution of Novel Choline-Binding Proteins to Adherence, Colonization and Immunogenicity of Streptococcus Pneumoniae. Mol. Microbiol..

[8] Makino S.-I., Yamamoto K., Murakami S., Shirahata T., Uemura K., Sawada T., Wakamoto H., Morita Y. (1998). Properties of Repeat Domain Found in a Novel Protective Antigen, SpaA, of *Erysipelothrix rhusiopathiae*. Microb. Pathog..

[9] Zhu W., Cai C., Wang Y., Li J., Wu C., Kang C., Sun X., Jin M. (2017). Characterization of Roles of SpaA in *Erysipelothrix rhusiopathiae* Adhesion to Porcine Endothelial Cells. Microb. Pathog..

[10] To H., Nagai S. (2007). Genetic and Antigenic Diversity of the Surface Protective Antigen Proteins of *Erysipelothrix rhusiopathiae*. Clin. Vaccine Immunol..

[11] Ingebritson A.L., Roth J.A., Hauer P.J. (2010). *Erysipelothrix rhusiopathiae*: Association of Spa-Type with Serotype and Role in Protective Immunity. Vaccine.

[12] Shiraiwa K., Ogawa Y., Nishikawa S., Eguchi M., Shimoji Y. (2017). Multiplex PCR Assay for the Simultaneous Detection and Differentiation of Clonal Lineages of *Erysipelothrix rhusiopathiae* Serovar 1a Strains Currently Circulating in Japan. J. Vet. Med. Sci..

[13] To H., Sato H., Tazumi A., Tsutsumi N., Nagai S., Iwata A., Nagano T. (2012). Characterization of *Erysipelothrix rhusiopathiae* Strains Isolated from Recent Swine Erysipelas Outbreaks in Japan. J. Vet. Med. Sci..

[14] Uchiyama M., Shimazaki Y., Isshiki Y., Kojima A., Hirano F., Yamamoto K., Kijima M., Nagai H. (2017). Pathogenic Characterization of *Erysipelothrix rhusiopathiae* Met-203 Type SpaA Strains from Chronic and Subacute Swine Erysipelas in Japan. J. Vet. Med. Sci..

[15] Morimoto M., Kato A., Kojima H., Akaike Y., Nogami K., Sasakawa C., Nagai S., To H. (2021). Serovars and SpaA Types of *Erysipelothrix rhusiopathiae* Isolated from Pigs in Japan from 2012 to 2019. Curr. Microbiol..

[16] Morimoto M., Kato A., Akaike Y., Nogami K., Ono H., Furusawa T., Kojima H., Sasakawa C. (2022). Comparative Study of the Phenotype and Virulence of Recent Serovar 1a, 1b, and 2a Isolates of *Erysipelothrix rhusiopathiae* in Japan. Vet. Microbiol..

[17] Uchiyama M., Yamamoto K., Ochiai M., Yamamoto T., Hirano F., Imamura S., Nagai H., Ohishi K., Horiuchi N., Kijima M. (2014). Prevalence of Met-203 Type SpaA Variant in *Erysipelothrix rhusiopathiae* Isolates and the Efficacy of Swine Erysipelas Vaccines in Japan. Biologicals.

[18] Ogawa Y., Ooka T., Shi F., Ogura Y., Nakayama K., Hayashi T., Shimoji Y. (2011). The Genome of *Erysipelothrix rhusiopathiae*, the Causative Agent of Swine Erysipelas, Reveals New Insights into the Evolution of Firmicutes and the Organism’s Intracellular Adaptations. J. Bacteriol..

[19] Ishida S., Tien L.H.T., Osawa R., Tohya M., Nomoto R., Kawamura Y., Takahashi T., Kikuchi N., Kikuchi K., Sekizaki T. (2014). Development of an Appropriate PCR System for the Reclassification of Streptococcus Suis. J. Microbiol. Methods.

[20] Nagai S., To H., Kanda A. (2008). Differentiation of *Erysipelothrix rhusiopathiae* Strains by Nucleotide Sequence Analysis of a Hypervariable Region in the SpaA Gene: Discrimination of a Live Vaccine Strain from Field Isolates. J. Vet. Diagnostic Investig..

[21] To H., Tsutsumi N., Akihiro T., Kamada T., Nagano T., Nagai S., Iwata A., Nunoya T. (2013). Protection of Immunized Mice and Swine to Challenge Exposure with *Erysipelothrix rhusiopathiae* Strains Obtained from Recent Swine Erysipelas Outbreaks in Japan. Acta Vet. Brno.

[22] Sawada T., Muramatsu M., Seko K. (1979). Response of Growth Agglutinating Antibody and Protection of Pigs Inoculated with Swine Erysipelas Live Vaccine. Nihon Juigaku Zasshi.

[23] Wang Q., Chang B.J., Riley T.V. (2010). Erysipelothrix rhusiopathiae. Vet. Microbiol..

[24] Takahashi T., Sawada T., Muramatsu M., Tamura Y., Fujisawa T., Benno Y., Mitsuoka T. (1987). Serotype, Antimicrobial Susceptibility, and Pathogenicity of *Erysipelothrix rhusiopathiae* Isolates from Tonsils of Apparently Healthy Slaughter Pigs. J. Clin. Microbiol..

[25] Shimoji Y., Osaki M., Ogawa Y., Shiraiwa K., Nishikawa S., Eguchi M., Yamamoto T., Tsutsui T. (2019). Wild Boars: A Potential Source of *Erysipelothrix rhusiopathiae* Infection in Japan. Microbiol. Immunol..

