# Supplementary material for: The Swine Erysipelas Vaccine SER-ME Effectively Protects Pigs against Challenge with the Erysipelothrix rhusiopathiae M203/I257 SpaA-Type Variant
Source: Vet Sci. 2022 Jul 26;9(8):382. doi: 10.3390/vetsci9080382 (PMC9332197; doi:10.3390/vetsci9080382)
Supplement: Supplementary file 1 [file vetsci-09-00382-s001.zip › vetsci-1808733-supplementary.pdf]

## Supplementary Materials

**Table S1.** Adverse events following the vaccination.

| Group No. | Pig No. | Immunization | The Primary Vaccination | | | | The Booster Vaccination | | | |
|---|---|---|---|---|---|---|---|---|---|---|
| | | | Red patch [*1] | Induration [*2] | Fever [*3] | Inactivity [*4] | Red Patch | Induration | Fever | Inactivity |
| 1 | 1 | Vaccinated (SER-ME) | − | − | − | − | − | − | − | − |
| | 2 | | − | − | − | − | − | + | − | − |
| | 3 | | − | − | + | − | − | + | − | − |
| | 4 | | + | + | − | − | − | − | − | − |
| 2 | 5 | Unvaccinated control | Not applicable | | | | | | | |
| | 6 | | | | | | | | | |
| | 7 | | | | | | | | | |
| | 8 | | | | | | | | | |
| 3 | 9 | Vaccinated (SER-ME) | + | + | − | − | − | − | − | − |
| | 10 | | + | − | + | − | − | − | − | − |
| | 11 | | + | − | − | − | − | − | − | − |
| | 12 | | − | − | − | − | − | − | − | − |
| 4 | 13 | Unvaccinated control | Not applicable | | | | | | | |
| | 14 | | | | | | | | | |
| | 15 | | | | | | | | | |
| | 16 | | | | | | | | | |

Adverse events following vaccinations were observed for 7 days. [*1] Red patch at the vaccination site. [*2] Induration by vaccination. [*3] Fever above 40.5 °C. [*4] Clinical signs of anorexia or depression. "−" shows no clinical sign and "+" shows adverse event.

**Table S2.** Detection of *E. rhusiopathiae* cells in organs of pigs.

| Group No. | Pig No. | Immunization | Challenge Strain | From Organs [*1] | | | | | | |
|---|---|---|---|---|---|---|---|---|---|---|
| | | | | Heart | Liver | Spleen | Kidney | Lung | Synovial Fluid | Mandibular Ltmph Nodes |
| 1 | 1 | Vaccinated (SER-ME) | Fujisawa (reference) | − | − | − | − | − | − | − |
| | 2 | | | − | − | − | − | − | − | − |
| | 3 | | | − | − | − | − | − | − | − |
| | 4 | | | − | − | − | − | − | − | − |
| 2 | 5 | Unvaccinated control | | + | + | + | + | + | + | + |
| | 6 | | | + | + | + | + | + | + | + |
| | 7 | | | + | + | + | + | + | + | + |
| | 8 | | | + | + | + | + | + | + | − |
| 3 | 9 | Vaccinated (SER-ME) | 2012 Miyazaki (variant) | − | − | − | − | − | − | − |
| | 10 | | | − | − | − | − | − | − | − |
| | 11 | | | − | − | − | − | − | − | − |
| | 12 | | | − | − | − | − | − | − | − |
| 4 | 13 | Unvaccinated control | | + | + | + | + | + | + | + |
| | 14 | | | + | + | + | + | + | + | + |
| | 15 | | | + | + | + | + | + | + | + |
| | 16 | | | + | + | + | + | + | + | + |

[*1] *E. rhusiopathiae* colonies from organ samples collected after the pigs' death including humane endpoint are shown. "−" shows no colony and "+" shows colonies on the agar plate.
